# Fast Benchtop Fabrication of Laminar Flow Chambers for Advanced Microscopy Techniques

**DOI:** 10.1371/journal.pone.0006479

**Published:** 2009-08-03

**Authors:** David S. Courson, Ronald S. Rock

**Affiliations:** Department of Biochemistry and Molecular Biology, The University of Chicago, Chicago, Illinois, United States of America; University of Strathclyde, United Kingdom

## Abstract

**Background:**

Fluid handling technology is acquiring an ever more prominent place in laboratory science whether it is in simple buffer exchange systems, perfusion chambers, or advanced microfluidic devices. Many of these applications remain the providence of laboratories at large institutions with a great deal of expertise and specialized equipment. Even with the expansion of these techniques, limitations remain that frequently prevent the coupling of controlled fluid flow with other technologies, such as coupling microfluidics and high-resolution position and force measurements by optical trapping microscopy.

**Method:**

Here we present a method for fabrication of multiple-input laminar flow devices that are optically clear [glass] on each face, chemically inert, reusable, inexpensive, and can be fabricated on the benchtop in approximately one hour. Further these devices are designed to allow flow regulation by a simple gravity method thus requiring no specialized equipment to drive flow. Here we use these devices to perform total internal reflection fluorescence microscopy measurements as well as position sensitive optical trapping experiments.

**Significance:**

Flow chamber technology needs to be more accessible to the general scientific community. The method presented here is versatile and robust. These devices use standard slides and coverslips making them compatible with nearly all types and models of light microscopes. These devices meet the needs of groups doing advanced optical trapping experiments, but could also be adapted by nearly any lab that has a function for solution flow coupled with microscopy.

## Introduction

Coupling of optical trapping with flow chambers, lab-on-a-chip, and other microfluidic devices has been accomplished by a number of groups[1]–[6] for two major purposes; cell sorting and manipulation[1]–[4] and making biochemical and biophysical measurements[5], [6]. Most of the cell sorting devices use traditional fabrication methods and have one optically uniform glass face while the channel walls and opposing face are made of a polymer such as polydimethylsiloxane (PDMS)[7].

Traditional microfluidic fabrication methods have several limitations. First, the fabrication method requires specialized equipment not readily available to many groups. Second, the PDMS face opposite the coverslip prevents some optical techniques that require accurate visualization through both sides of the device. One such technique is optical trapping with precision force and position measurements, because the PDMS layers are of non-uniform density and distort the wavefront of the required detection lasers in unpredictable ways. This can make experiments such as the study of molecular motor stepping[8], [9] or forced unfolding[10] problematic in such devices. Since buffer conditions can change these behaviors, being able to quickly change the buffer conditions while studying the same molecules could be useful but remains illusive.

As a result of these difficulties most trapping experiments that require precision measurements are performed using chambers made of a slide and coverslip linked by a piece of double sided tape and sealed with vacuum grease[11]. When multiple conditions are required, multiple independent experiments are run. While very effective these devices have limitations as well. Experiments using sealed sticky tape devices require premixing all components, so unwanted component self-association prior to visualization is a problem. It is also difficult to make complex geometry devices using tape flow cells. Further, coupling tape devices to flow often requires drilling through glass and attaching ports, which can interfere with optical components such as oil condensers[12].

For biochemical and biophysical measurements with flow other devices have been produced. The Kowalczykowski group has coupled optical trapping and flow technology to generate a platform for performing fluorescence microscopy measurements on proteins bound to a single DNA molecule[5]. However these devices are made from etched glass, which requires special facilities to fabricate and have not been, to our knowledge, coupled to high-resolution detection systems.

Recently the Wuite group developed a system to couple flow and optical trapping with high-resolution position and force detection[6] to examine the single molecule behavior of DNA binding proteins. They report using parafilm to construct channels. In our hands uniform parafilm adherence was problematic so these devices tended to leak. We also had difficulties incorporating fluid ports that would not interfere with our optics. Our research requires a device of this type that prevents reagent mixing and self-assembly but allows trapped particles to be transported into different environments. Here we present a simple manual method for flow chamber fabrication that can be preformed on a benchtop and is fast, inexpensive, requires no glass drilling, produces devices that are reusable and meet the requirements of advanced optical trapping assays, including all-glass optical faces, and a radial arrangement of tubing connections that allows us to use oil-immersion condensers and objectives.

## Methods

### Device Fabrication

**Figure 1:**
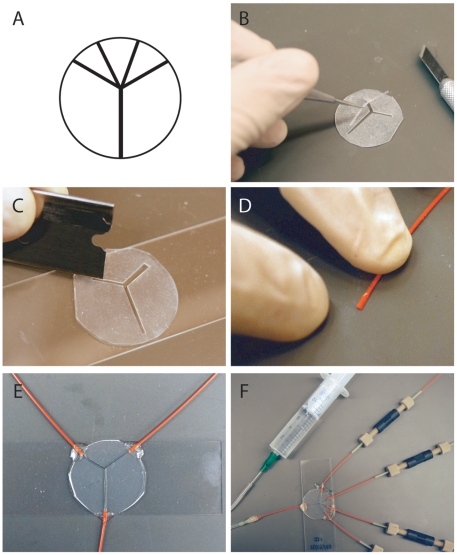
Fabrication and use of devices. A) Scale pattern of a four input device made to fit a 25 mm round coverslip. B) Device body of a two input device being prepared. The channel pattern is cut out of the body but the channel entrances are left intact so the device remains one solid piece during the process of cleaning and adhering to the slide. C) Channel entrances are cut open with a razor blade. A scraping motion is needed to remove the piece once cuts are made. D) Pieces of PEEK tubing are cut and one end is flattened to fit into the device. E) Tubing is attached using epoxy. The device is complete at this point. F) Example of a functioning four input device attached via HPLC connectors to a reservoir and to a priming syringe via silicone tubing. These linkages can be modified to fit other systems.

Our flow chamber design uses a single flat sheet of silicon rubber, which is cut by hand into the desired shape. The advantage of using commercially available silicon rubber sheets is that they have a uniform thickness. Moreover, they are easily cut by hand and manipulated, since they only adhere once activated by oxygen plasma. We cut out a piece of silicon rubber (McMaster-Carr, 87315K63) of the same dimensions as the coverslips that will be used (25 mm round) (see Supplemental Material S1 for more detailed illustrated step-by-step assembly and usage protocols). Next the desired pattern is cut into a silicon rubber sheet using sharp razors (Pattern in Figure 1a). Channel entrances should be left intact at this point so that sheet remains in one piece until after it is joined to the slide (Figure 1). The cut sheet constitutes the device body.

The microscope slide and the device body should be rinsed with pure, deionized water and dried to remove large debris. Plasma clean the slide and device body to functionalize the surfaces (acid washing will also work eliminating the need for a plasma cleaner[13]). Once cleaned press the device body onto the slide. The bond is permanent. Cut channel entrances open with the razor.

Next plasma clean the device complex and coverslip and press them together. This produces a sandwich of rubber with channel openings between the slide and coverslip. Thin wall PEEK tubing (Upchurch, 1569) is then connected to the device. Compress tubing at one end and slide into channel entrances. Seal the joint between the device and the tubing with quick drying epoxy. Once dry the device is complete and can be hooked up to a flow driving system via HPLC adaptors. All devices should be tested for leaks and correct flow characteristics before use in experiments.

### Device Testing

Since the devices are not precision cut or cast, there will be variance from device to device. Thus, if very specific or complex flow characteristics are required this technique may not be suitable. Additionally, each flow cell should be characterized individually. For example, in multiple input laminar flow cells, if there is variance in the structure of the input channels or the length of the tubing leading from the reservoir the width and flow rate of each lane in the main channel may vary slightly. Even with these limitations for many applications this method is sufficient.

Flow rates can be measured in several ways. For high flow rates [>1 ul/sec] driving a set amount of fluid through the device and measuring the time required to pass a given volume is an effect method. For extremely slow flow rates, observing the motion of particles such as fluorescent beads as they transit a known distance as visualized by the microscope can give an accurate estimation of flow rate. It is important to note that flow rates are fastest in the center of the channels and slower near surfaces. If driving flow by gravity it is important to remember that rates will drop as the column heights balance. Once at a desired flow rate, the water column height must be routinely adjusted to maintain the flow rate.

### Device Cleaning

Since these systems are entirely composed of PEEK, silicon rubber and glass they are very chemically inert. As such they can be cleaned very rigorously allowing them to be reused without fear of contamination. We rinse the systems sequentially with water, 1 M NaOH, EtOH then finally water again (see Supplemental Material S1 for details). This cleans all internal surfaces.

### Myosin Motility Assay

This assay was performed in two ways: in a flow chamber and a series of sealed chambers (the current standard method). Data in panels 3b and 3c were collected on different days but used the same motor preparation and solution stocks. Incubation times and all other variables were kept as similar as possible.

For the flow chamber experiments, a four input laminar flow chamber was used. The device was primed with water by injecting it into the device via the output port using a syringe. All setup solutions are added in this way. This eliminates bubbles and insures all inputs are exposed to the same conditions. Anti-GFP antibody (QBiogene, 50 ng/µl) was added into the device to coat all of the internal surfaces[14]. BSA was then added as a blocking agent (1 mg/ml). GFP tagged Myosin VI heavy meromyosin (HMM) dimer [14] was added followed by filamentous actin (chicken skeletal muscle, 100 nM [15]), and then assay buffer (25 mM imidazole, pH 7.5, 25 mM KCl, 1 mM EGTA, 4 mM MgCl2, 10 mM DTT, 0.86 mg/ml glucose oxidase, 0.14 mg/ml catalase, 9 mg/ml glucose) with zero ATP. The microscope was focused and a desirable field of view was found. Assay buffers with desired ATP concentrations (0, 0.1, 1, 2 µM) were loaded into the input reservoirs. Movies were then recorded for the zero ATP condition. The valve to the 0.1 µM ATP containing solution was then opened, a sufficient replacement volume was allowed to flow through and a movie was recorded. Data in panel 3b were recorded with the flow turned off. Data in panel 3c were recorded with flow off then with flow on. This process was repeated for each ATP solution. Data was recorded on a home built total internal reflection fluorescence (TIRF) microscope with Andor iXon camera. Movies were analyzed using ImageJ.

### Optical Trapping Assay

All experiments were performed on a home built fluorescence and optical trapping microscope.

#### Power Spectra

A two input device was used. The system was primed as described above. 1 µm beads were loaded into one input reservoir, and assay buffer into the other. Beads were flowed into the device and a single bead was trapped. Flow rate was approximately 1 µL/sec. The trapped bead was moved into the buffer channel and the bead position was recorded at 10 kHz, without a lowpass antialias filter. Flow was then turned off and the power spectrum was repeated with the same bead. Power spectra were calculated using the Igor Pro software package.

#### Dumbbell

A four input device was used. BSA blocking solution was pumped into the output port of the device as described above to prime the device and block the surface. 1 µm neutravidin coated beads (biotinylated beads from Invitrogen incubated in a neutravidin solution and blocked with BSA) were loaded into one input reservoir, TMR-phalloidin (Sigma-Aldrich) stabilized actin filaments with ten percent of the monomers labeled with biotin were loaded into the second input reservoir, and buffer was loaded into the third and fourth. The solutions were allowed to flow into the device. Beads were trapped then the stage was moved so that those beads transited into the actin containing channel. Once an actin filament was captured (by flowing against a bead) the stage was again moved so the beads and captured filament moved into the buffer channel where the dumbbell was assembled. The bead with the attached filament was placed upstream. Buffer flow extended the filament. The second bead was then moved into position at the free end of the extended filament and the bond was formed.

## Results

Here we present data from several experiments that show the broad applicability and high degree of functionality of these devices. This data shows that these devices are functional for a broad range of microscopy experiments including sensitive optical trapping experiments.

### Device Characteristics

**Figure 2:**
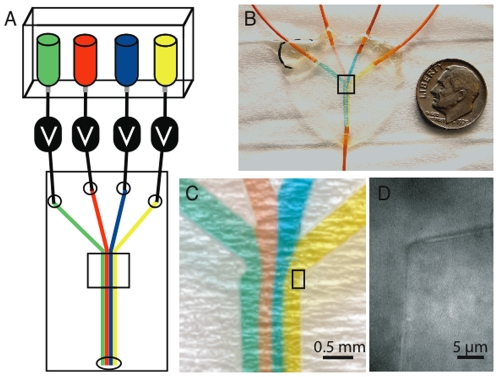
Device Details and Flow Characteristics. A) Diagram of basic flow chamber design. Solutions are stored in reservoirs and must pass through a solenoid valve to reach the flow chamber. Solutions can be added one by one or multiple at once. When multiple solutions are added they flow in separate laminar flow lanes. This behavior is preserved regardless of the number of solutions entering the flow chamber. All the solution flows out through a single output port. B) Picture of a four input flow chamber with four solutions flowing. Each input has a different colored water solution flowing through it to demonstrate laminar flow characteristics of the device. The device is made with a standard microscope slide and 25 mm round #1.5 coverslip. Orange PEEK tubing is connected to each input and the output with epoxy. The output tube leads to a collection reservoir mounted on an adjustable height platform to allow control of the flow rate by altering the height of the water column difference between the input reservoirs and the output tubing end. C) Blow up of flow chamber from highlighted area in panel B, showing laminar flow characteristics. Small defects such as the one on the leftmost corner do not significantly affect the downstream flow profile. D) Blow up from inside the highlighted area in panel C. Bright field image of a feature in the device. This image shows the corner where one of the input channels joins the main channel. The scale bar is set over the rubber section while the figure letter is set over the input channel solution. Even though these devices are cut by hand they typically have very fine and clean features so solution flow is not perturbed.

Devices were fabricated using main channel widths of 0.3 up to 2 mm, input channel widths of 0.5 to 1.0 mm, and between 1 and 5 inputs. All devices produced laminar flow, where each solution in the main channel remained separate and only mixed via diffusion (Figure 2). Devices were tested over a range of flow rates from greater than a 1 milliliter per second to rate of below 1 microliter per minute.

### Myosin Motility Assay with Multiple ATP Concentrations

**Figure 3:**
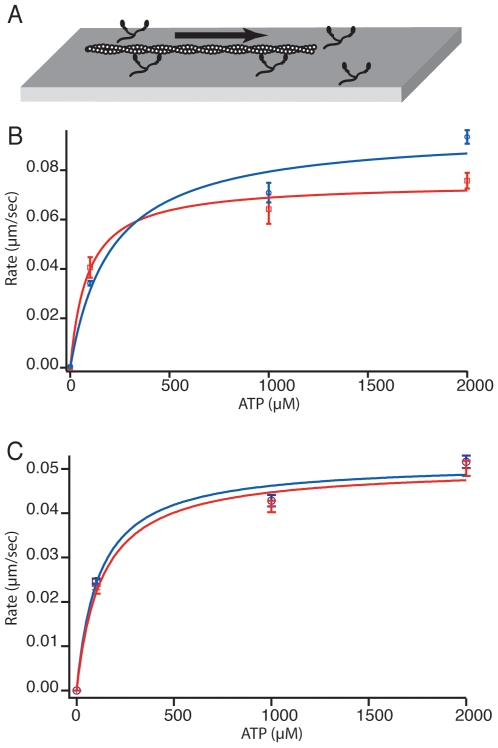
Myosin VI Motility. A) Motility assay cartoon. Myosin VI motors are immobilized on a coverslip surface. Actin filaments land on the surface and are then propelled by the motor proteins. B) Graph of motility rate verses concentration of ATP. Data in red (squares) indicates experiments performed in separate sealed chambers. Data in blue (circles) indicates a single experiment performed in a flowcell device where a desired ATP solution was flowed into the chamber, flow was stopped and data was recorded for each concentration. Michaelis-Menton fit for each data set in corresponding colors. Fits are similar though some difference is seen at 100 µM and 2 mM. Fit parameters for sealed chambers: Vmax = .075±.0040 µm/sec, Km = 88±25 µM. Fit parameters for flowcells: Vmax = .096±.0096 µm/sec, Km = 206±95 µM. C) Graph of motility rate verses concentration of ATP. Comparing rates obtained in a single flow chamber with flow on (red cross) and off (blue circles). Flow rate used was approximately 1 µl/sec. The fits are nearly identical, indicating surface shear experienced by the motor when flow is on had no measurable affect of motor behavior. Fit parameters with flow: Vmax = .051±.0030 µm/sec, Km = 129±38 µM. Fit parameters without flow: Vmax = .051±.0031 µm/sec, Km = 114±35 µM. Indeed, variation from day-to-day or sample-to-sample is far higher than variation caused by flow shear, as is evident by the difference in behavior between samples in panel B and panel C.

As a proof-of-principle experiment these devices were used to perform myosin gliding filament assays under varying ATP concentrations using TIRF microscopy. Myosin VI (as is true of all myosins) shows an ATP concentration dependent motility rate [16], [17]. The device was coated with myosin VI before actin filaments were added. Some filaments were bound by the motors at the coverslip surface and immobilized in the absence of ATP. A series of motility buffers with increasing ATP concentrations (0, 0.1, 1, 2 µM) was then allowed to flow into the flow chamber and movies were recorded at each ATP concentration, with and without flow. The addition of ATP caused the motors to move (Figure 3). The motility profiles were similar between the two assays.

There are several advantages to the flow chamber method. This method allowed us to assess the ATP-dependent motility of a single population of motors, rather than preparing a different slide to analyze each ATP concentration. This prevents slide-to-slide variation, making results easy and reliable to interpret. Open chamber devices (sticky tape) can dry out during the course of an experiment as happens if inadequately sealed, changing ATP concentration. The flow chambers presented here do not share this limitation. Finally, continuous solution flow can also be used to prevent the accumulation of ADP in the chamber, which could lower the apparent motility rate of the motor. The shear induced by flow does not appear to affect myosin VI motility but it should be considered if assaying other motors in the presence of flow.

### Optical Trapping Assays

To demonstrate that optical trapping, manipulation, and position detection is practical with these devices two assays were performed. 1) We trapped 1 µm polystyrene beads and measured power spectrums with and without flow. 2) We assembled actin dumbbells and manipulated them. The first experiment uses optical trapping, bright field microscopy and laser position detection. The second uses optical trapping, bright field and epifluorescent microscopy.

#### Power Spectra

**Figure 4:**
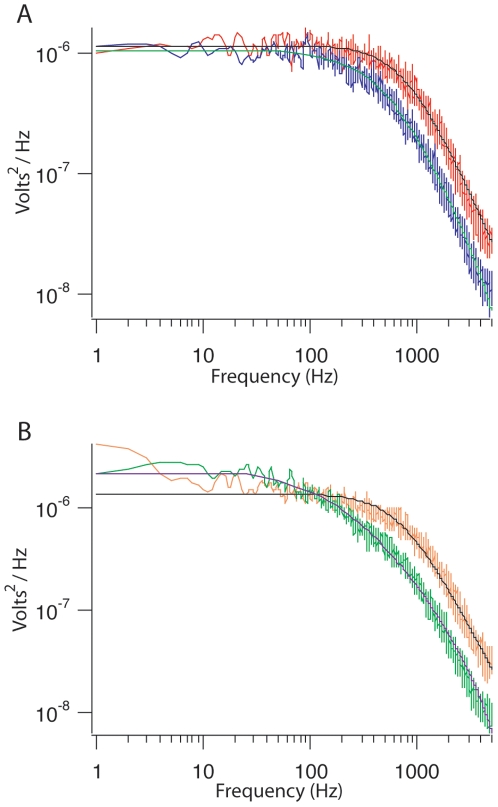
Power Spectra. A) Representative power spectrum for a trapped bead in a flow chamber with flow turned off. A spectrum is taken in X and Y coordinate systems. X is shown in red, Y in purple. Lorentzian fits are overlaid on the each spectrum. The black fit is for X, the blue fit is for Y. The bead corner frequency for X = 781 Hz, for Y = 472 Hz. B) Power spectrum data for the same bead after the flow was turned on at a rate of 1 µl/sec. Flow is in the X direction. X is shown in orange, Y in green. The black fit is for X, the purple fit is for Y. The bead corner frequency for X = 697 Hz, for Y = 226 Hz. Introduction of flow causes some low frequency noise in both spectra and shifts the corner frequency of the Y power spectra with respect to the no flow. X spectra are very similar for each condition.

A bead was trapped under flow of 1 µL/sec flow rate. A power spectrum was recorded [8], [18], [19]. Then flow was stopped and another power spectrum was measured. The power spectra are nearly indistinguishable in the X direction (the direction of flow) (Figure 4). First this demonstrates that trapping is indeed possible in these devices. Second these devices are functional for position detection and force measurements when the system is under flow and when flow is stopped. This creates a dynamic environment in which to design and perform experiments.

#### Dumbbells

**Figure 5:**
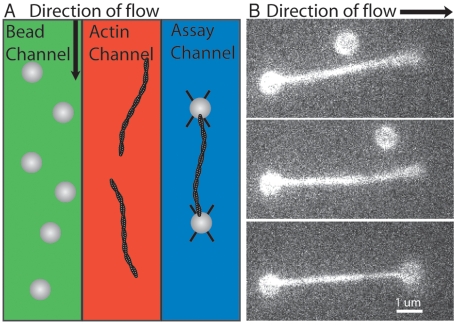
Dumbbell Assembly. A) Diagram of method for assembling an actin dumbbell while under flow. Beads are trapped in the bead channel, the stage is moved transferring the trapped beads into the actin channel where one filament is attached to one of the beads. The stage is moved again transferring the trapped beads and actin filament into the assay chamber where a dumbbell is formed and manipulated. B) A trapped 1 µm neutravidin coated bead with and attached biotinylated filament is held in an optical trap. Flow keeps the filament aligned down stream. A second trapped bead is then moved into position near the free end of the filament. When the bead and filament touch they become tightly linked. Introducing the components in separate channels has the advantage that the beads and filaments are isolated and do not spontaneously assemble into undesired aggregates.

For many actin based optical trapping experiments, such as analysis of myosin motors stepping, forming an actin dumbbell is required [20], [21]. A dumbbell is an actin filament with each end connected to beads held in separate optical traps. Here we trapped two beads under flow, attached an actin filament to one of those beads under flow, then moved the second bead into position downstream behind the trapped filament to form the dumbbell (Figure 5, Supplemental Movie S1). The trapped filament can be manipulated and used for desired assays.

Unlike traditional methods where beads and actin filaments are mixed together in extremely low dilutions and the user must find and assemble them (a process which can be time sensitive), using the flow based method present here each component is added in isolation and can be assembled extremely quickly (as fast as 13 seconds for a dumbbell in our hands). Further, since a buffer channel is present which lacks any other components, these structures can be manipulated without concern for accidental addition of unwanted components (another bead or actin filament sticking to the trapped dumbbell for example). This ability increases the percentage of experiments that are successful under most circumstances. Additional flow lanes can be added to allow for addition of other components to the trapped scaffold, as was demonstrated by the Wuite group's DNA and H-NS experiments[6].

## Discussion

### Design and Potential Applications

Beyond the ability to perform specialized optical trapping assays these devices have other important virtues. The fabrication technique reported here requires no glass drilling, no training, and little to no specialized equipment (plasma cleaner is recommended). Devices are constructed using standard microscope slides and coverslips making them compatible with nearly all light microscopes. The assembly protocol is general enough that any glass coverslip can be accommodated, allowing for matching of coverslips to objectives for optimum results and making design of devices of different dimensions simple. The slide can even be replaced by a second coverslip to accommodate dual objective optical trapping microscopes. The fluid volume of the devices is also flexible ranging from 30 down to 3 microliters depending on design (see Supplemental Material S1 for ways to lower device volume). After most usages devices can be cleaned and reused. Because of the uniform nature of the silicone rubber sheets used to make these devices, when the devices are properly assembled the coverslips lay extremely flat allowing for minimal focal drift as the sample is moved. All fluorescence imaging characteristics seem to closely mimic those of sealed sticky tape flow cells, including low oxygen permeability yielding long fluorescence lifetimes and the ability to clearly resolve single molecules.

These devices do carry inherent limitations, which should not be overlooked. Since channels are cut by hand variation from device to device will occur. For some applications this will require that each device be tested before use. Complex multilayer applications such as those requiring pneumatic microvalves [22], [23] cannot be adapted to work with these devices. Finally device volumes are typically around 10 microliters and require a reservoir to generate flow, so applications that use very small volumes are not practical.

We see several areas in which these devices could be immediately applicable. The flexibility in device design, low cost, and the ease of construction should allow them to be adapted by groups in many fields. Laminar flow chambers, from single to many inputs, can be made exactly as described here. Coupling valves to the fluid handling system allows for many possibilities from side-by-side laminar flow to iterative additions of different solutions.

Coupled with valves these devices allow for rapid and repeated changes in buffer conditions. With computer-controlled valves, experiments or condition screens could be automated saving researchers time and effort. Our system uses commercially available solenoid valves (Lee Company) to control inputs.

Most current optical trapping experiments use sealed chambers. This necessitates mixing all experimental components together before starting the experiment. If these components interact with each other in solution then it becomes difficult to control and study these interactions. Devices like these allow for iterative assembly of complex systems, opening up new avenues of exploration for single molecule scientists[6]. As our knowledge increases study of more complex systems becomes necessary. These devices should serve as a useful tool for scientist attempting to do that.

## Supporting Information

Material S1 Detailed assembly and usage protocols.
(2.61 MB DOC)

Movie S1 Making a Dumbbell. Construction of an actin dumbbell
(2.05 MB MOV)
