# Supplementary material for: Fast Benchtop Fabrication of Laminar Flow Chambers for Advanced Microscopy Techniques
Source: PLoS One. 2009 Aug 3;4(8):e6479. doi: 10.1371/journal.pone.0006479 (PMC2714461; doi:10.1371/journal.pone.0006479)
Supplement: Material S1 — Detailed assembly and usage protocols. (2.61 MB DOC) [file pone.0006479.s001.doc]

**Supplemental Materials S1:**

Required Materials:

| **Description** | **Company** | **Part Number** |
| --- | --- | --- |
| Silicone rubber sheets (.020 inch thickness) | McMaster-Carr | 87315K63* |
| PEEK Tubing 1/32 OD x .020 ID | Upchurch | 1569* |

*Thinner sheets and smaller tubing can be used. This will also lower the solution volume required and change flow characteristics.

Other needed items: Epoxy, slides, coverslips, razor blades, forceps, Teflon block or non-stick pad, bath sonicator, plasma cleaner or acid bath, HPLC adaptors, solution reservoirs and flow drive system. We also recommend valves to control each input separately. We use solenoid valves from the Lee Company, with driver electronics as suggested in their product literature.

Detailed Protocol:

Making the Device

Important Note:

• This is a basic protocol. Patterns and some conditions will have to be modified for different purposes.

• Since each device is fabricated by hand, each design must be thoroughly tested to insure it has the desired flow characteristics.

• Wear gloves. Oils from your hands will prevent proper adhesion of the rubber to the

glass.

Making Device

| **Step** | **Picture** | **Detail** |
| --- | --- | --- |
| 1 | 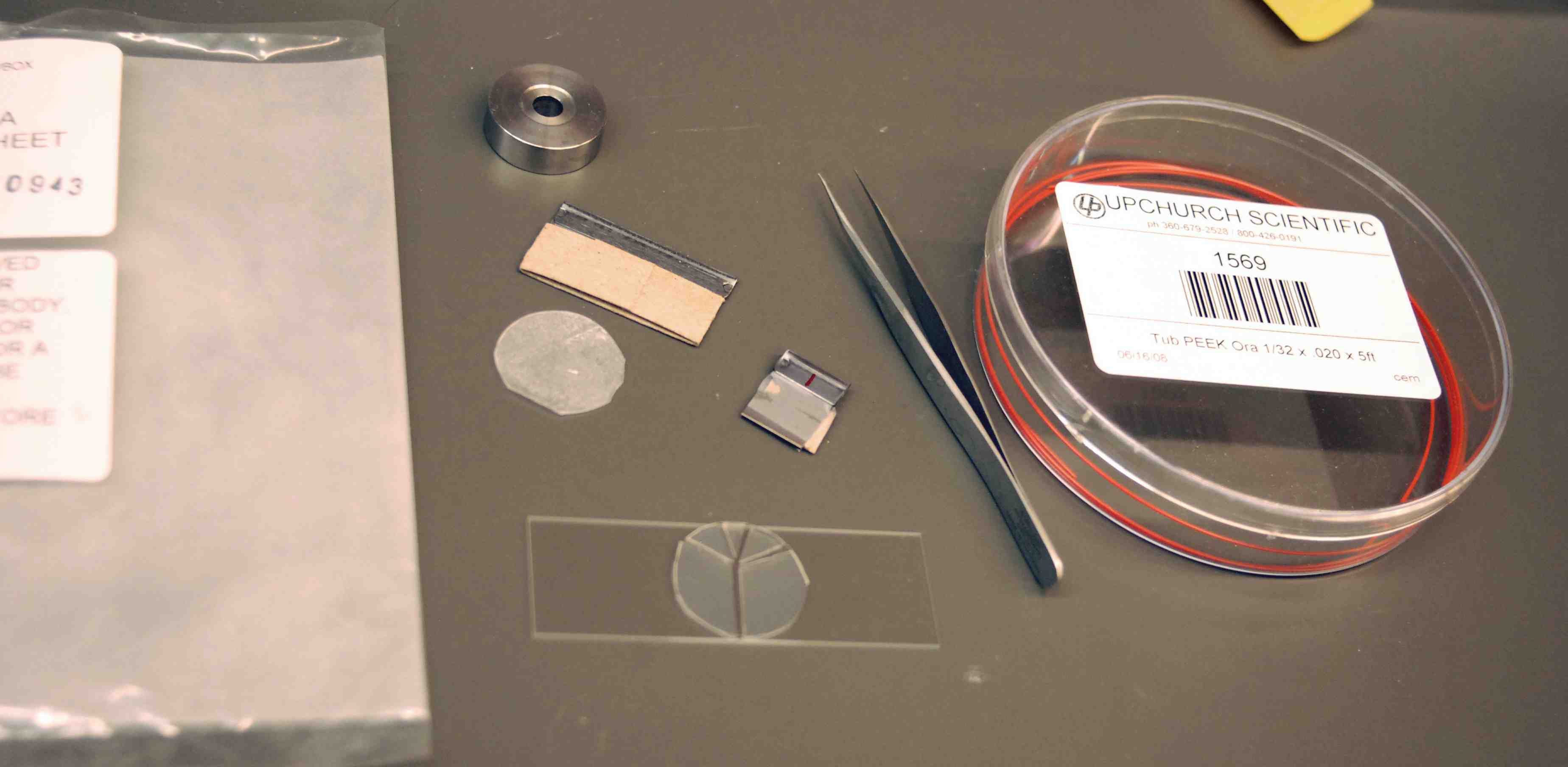 | Decide on a size of the device, including slide and coverslip dimensions. |
| 2 |  | Make a scale design of the device on paper. In most cases this pattern should be the same size as the coverslip. |
| 3 | 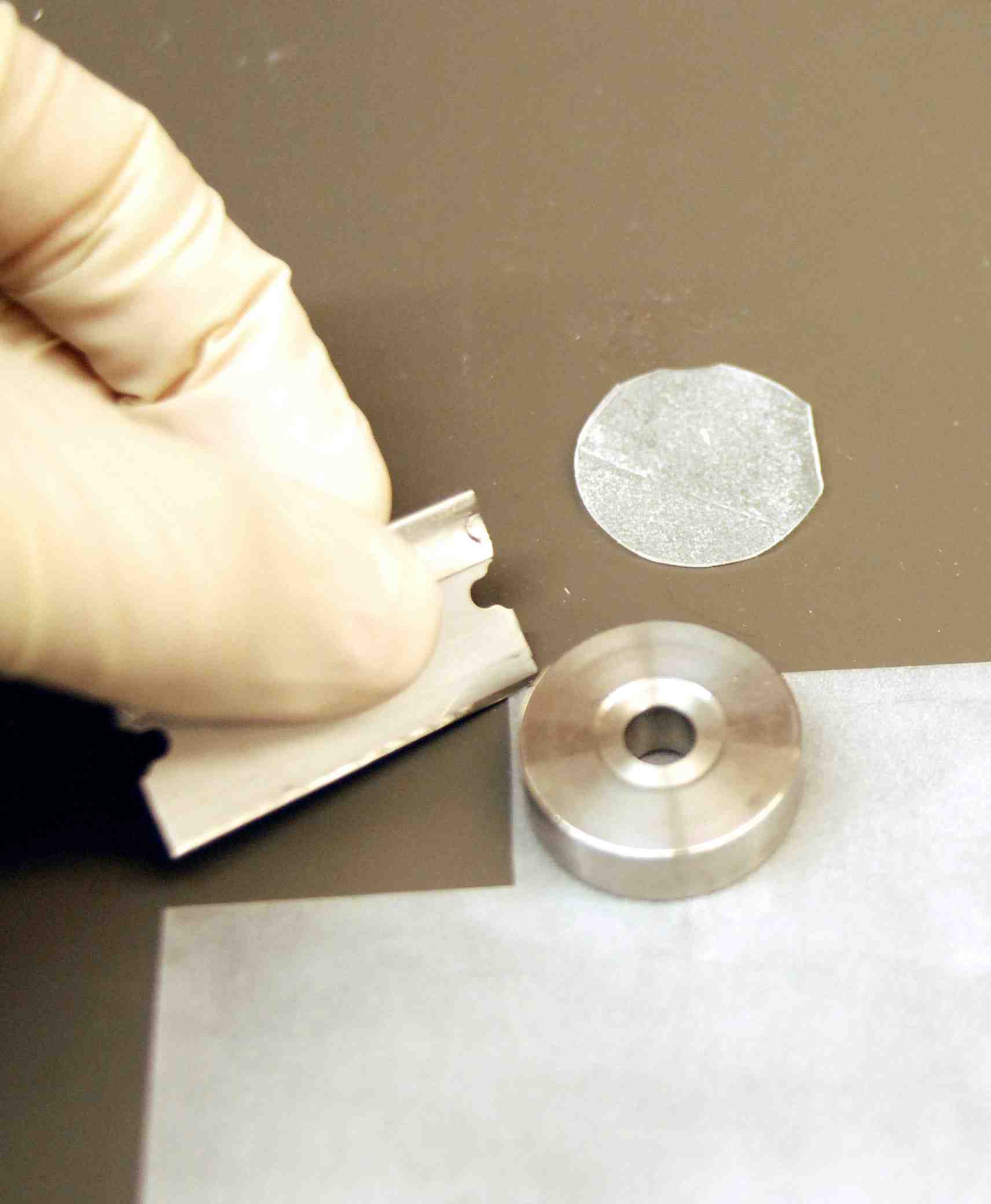 | Cut the rubber sheet to match the outer dimensions of your device. |
| 4 |  | Rinse the excess powder off the sheet. |
| 5 | 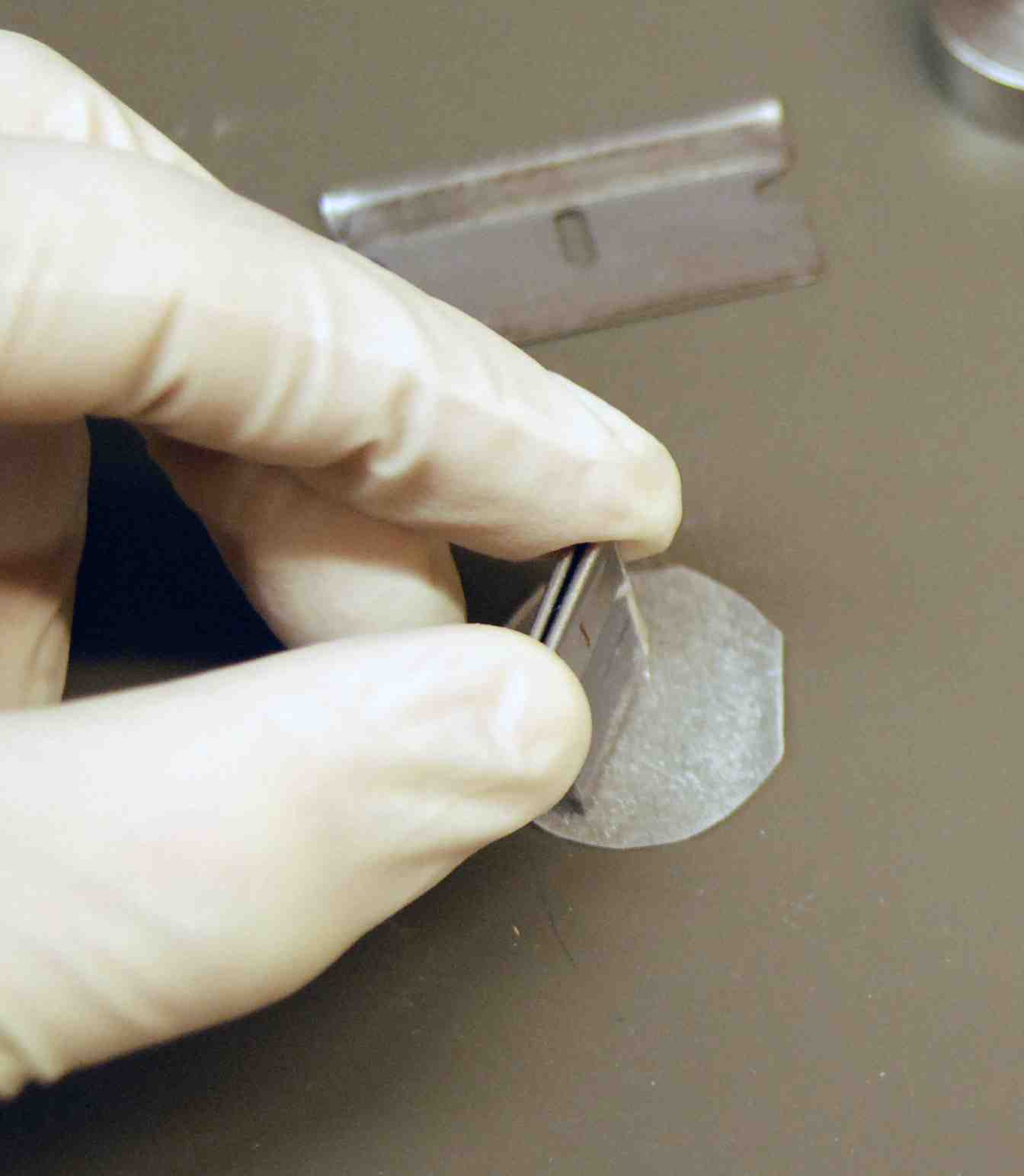  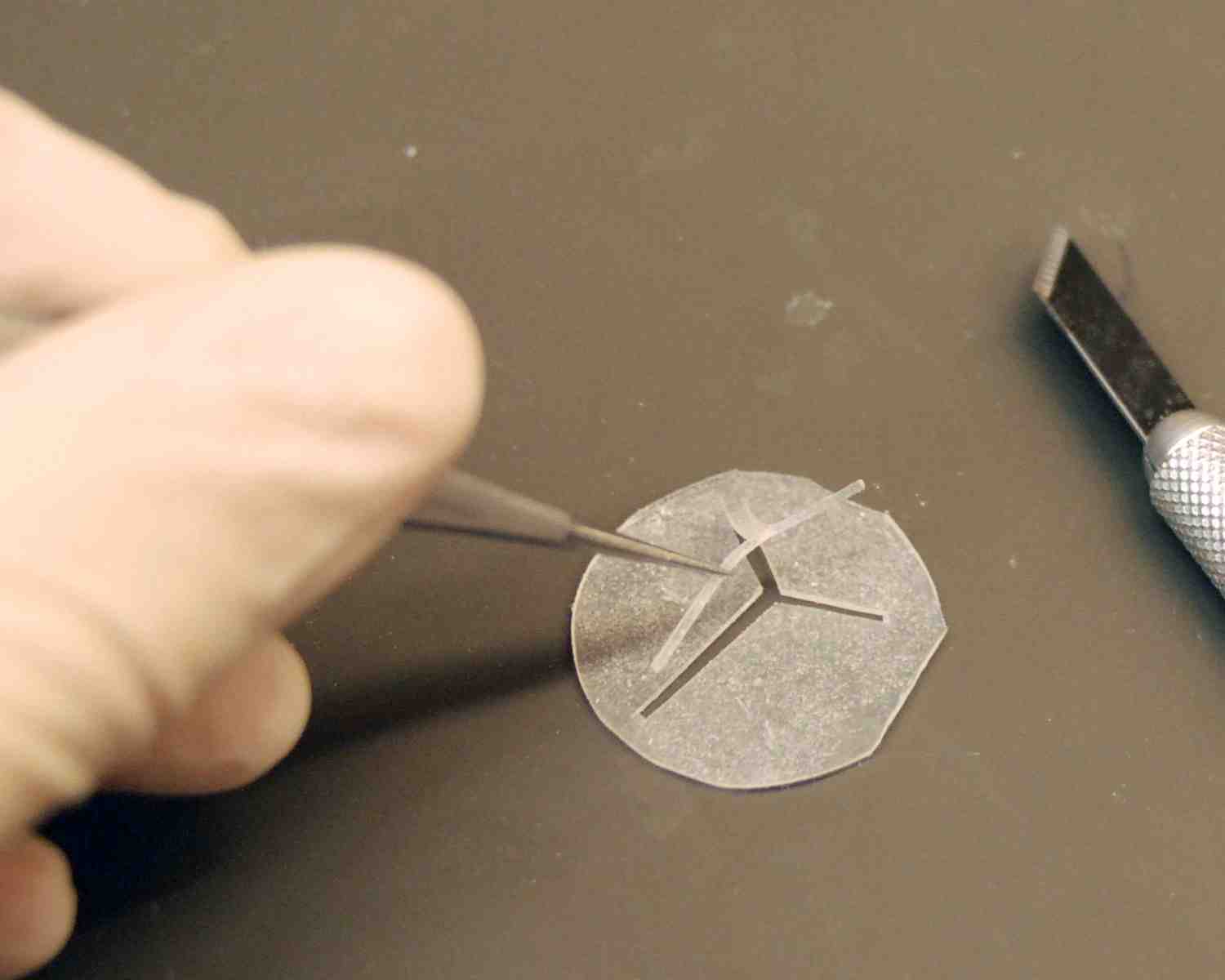 | Place the pattern under your sheet and cut according to the pattern. Leave the channel entrances intact. They will be cut out later. Razor blades taped together are ideal for this purpose, but cutting the sheets is easiest with downward pressure rather than dragging or sawing with the blade. For this reason having razors of a specific length is ideal. Razor blades can easily be cut to any length with a sheet metal cutter. Dragging or sawing with the blades will work but requires more care to prevent defects. |
| 6 |  | From this point on handle the sheet (device body) as little as possible, using forceps when possible. (Optional) Sonicate the device body, coverslip and slide in a clean bath sonicator for 2-5 minutes to clean and break off any loose debris. |
| 7 |  | Dry with isopropyl alcohol or N_2_ gas. Pieces must be completely dry before proceeding. |
| 8 | 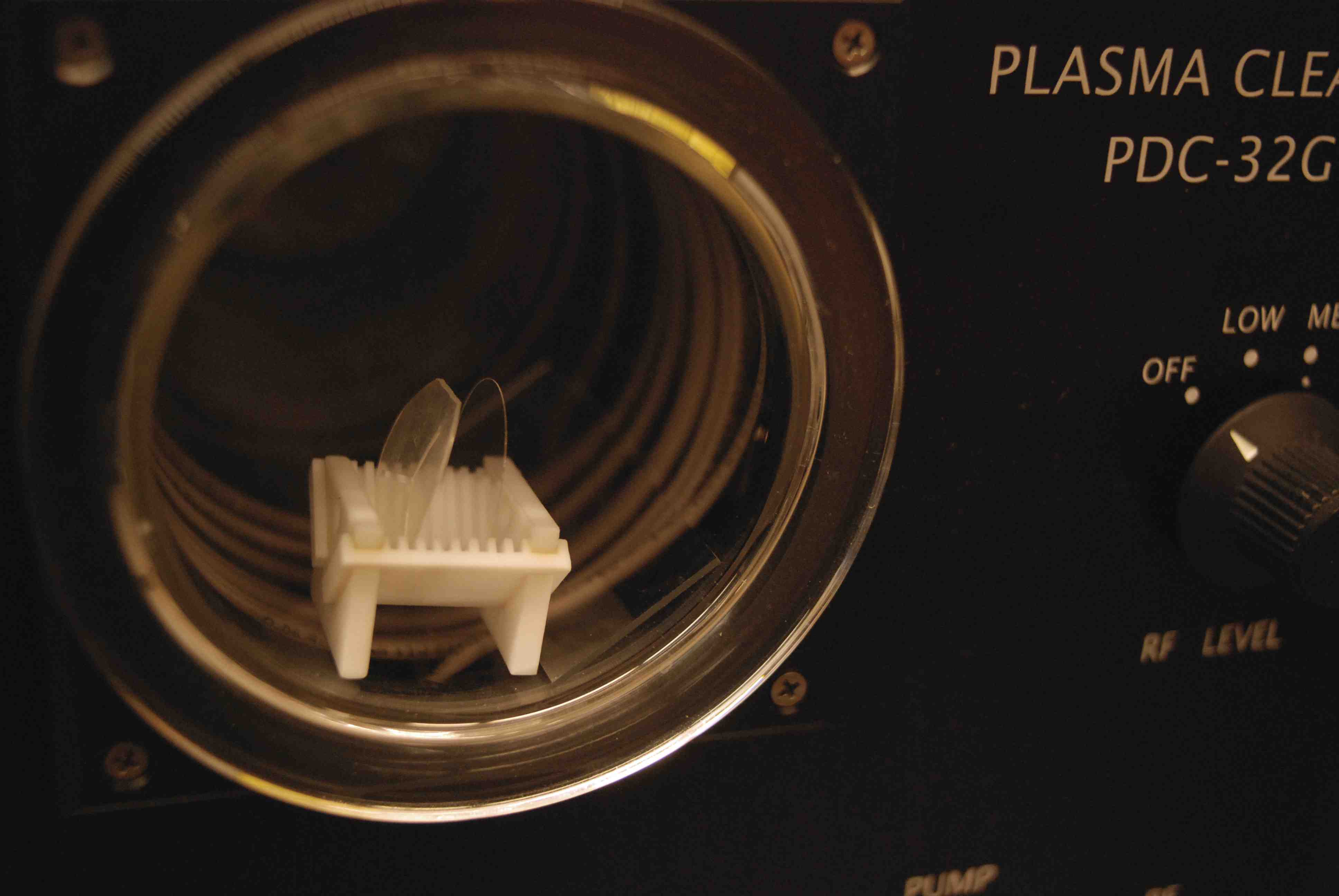 | Plasma clean all the pieces for 30 seconds to 2 minutes. The Teflon racks are good for preventing the device body from sticking to the plasma cleaning chamber or the other pieces. (Note: All plasma cleaning steps can be replaced by an acid wash step as described by Campbell and Groisman. However to do this we recommend washing the coverslip, slide and device body all at once to save time, and then handling carefully.) |
| 9 | 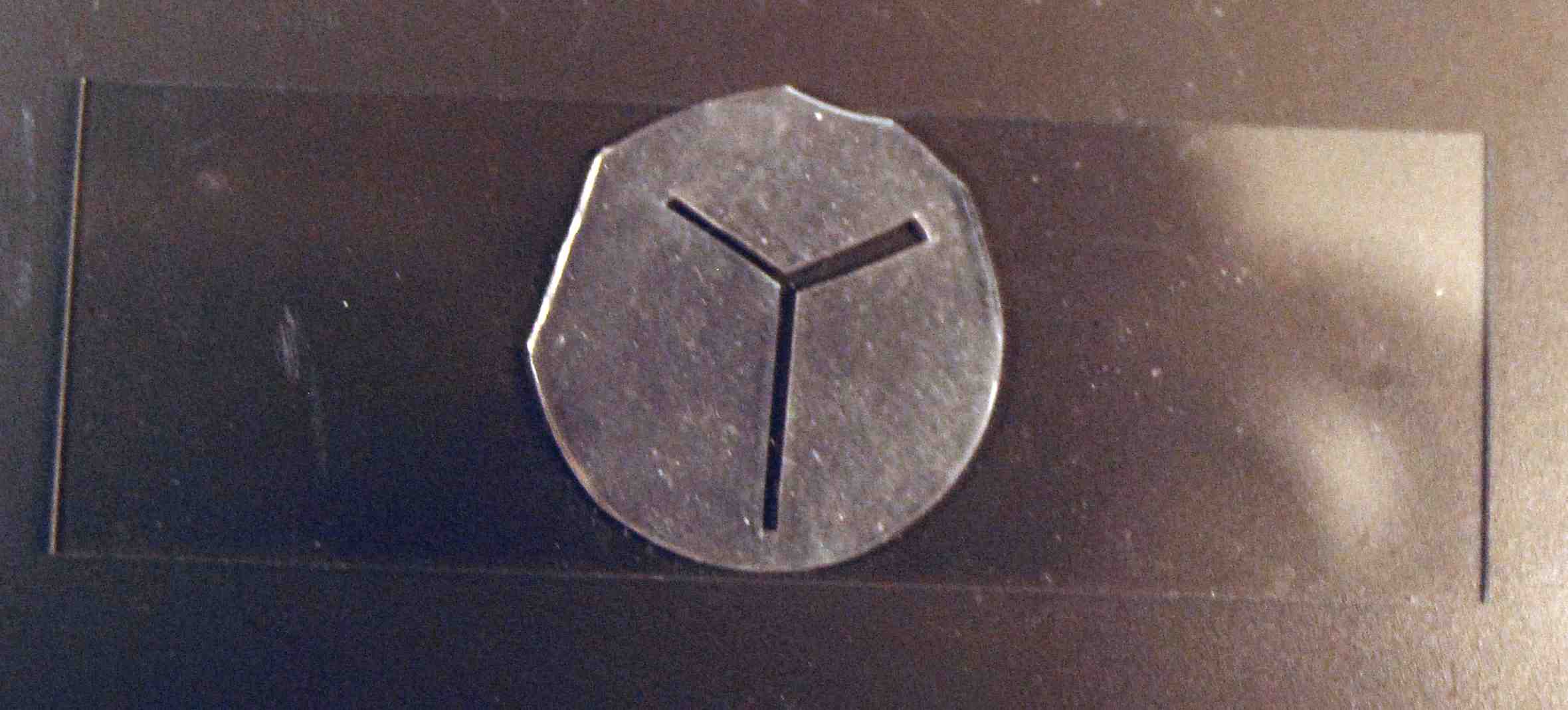 | Press the device body onto the slide. Try to lay it down in one fluid motion and smooth out all bubbles and creases. |
| 10 | 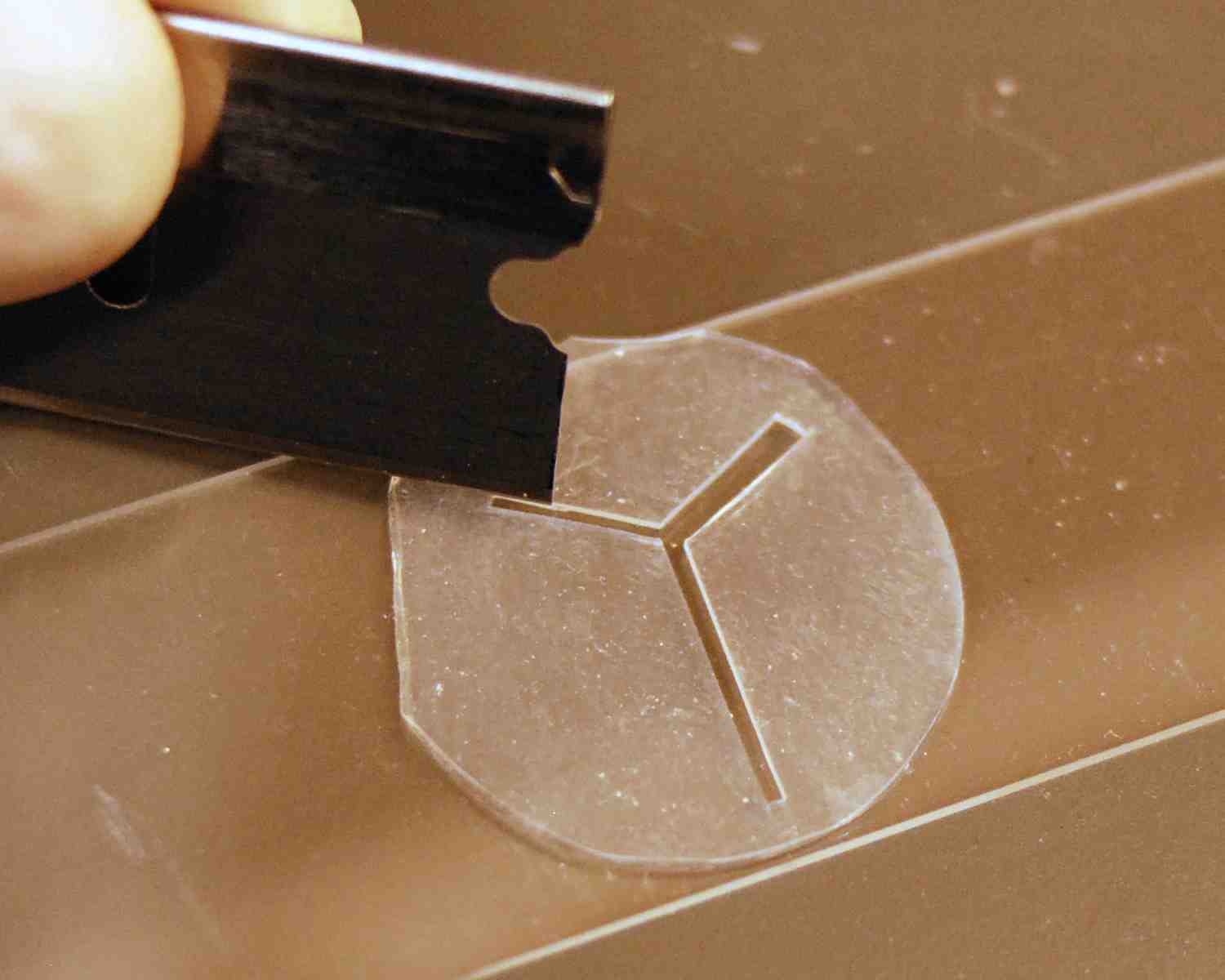 | Using a razor blade cut open the channel entrances. The rubber will adhere very tightly to the glass, so once the channel openings are cut you may have to scrape the unwanted piece of rubber out with the razor blade. |
| 11 |  | Plasma clean the device complex for an additional 30 seconds. |
| 12 | 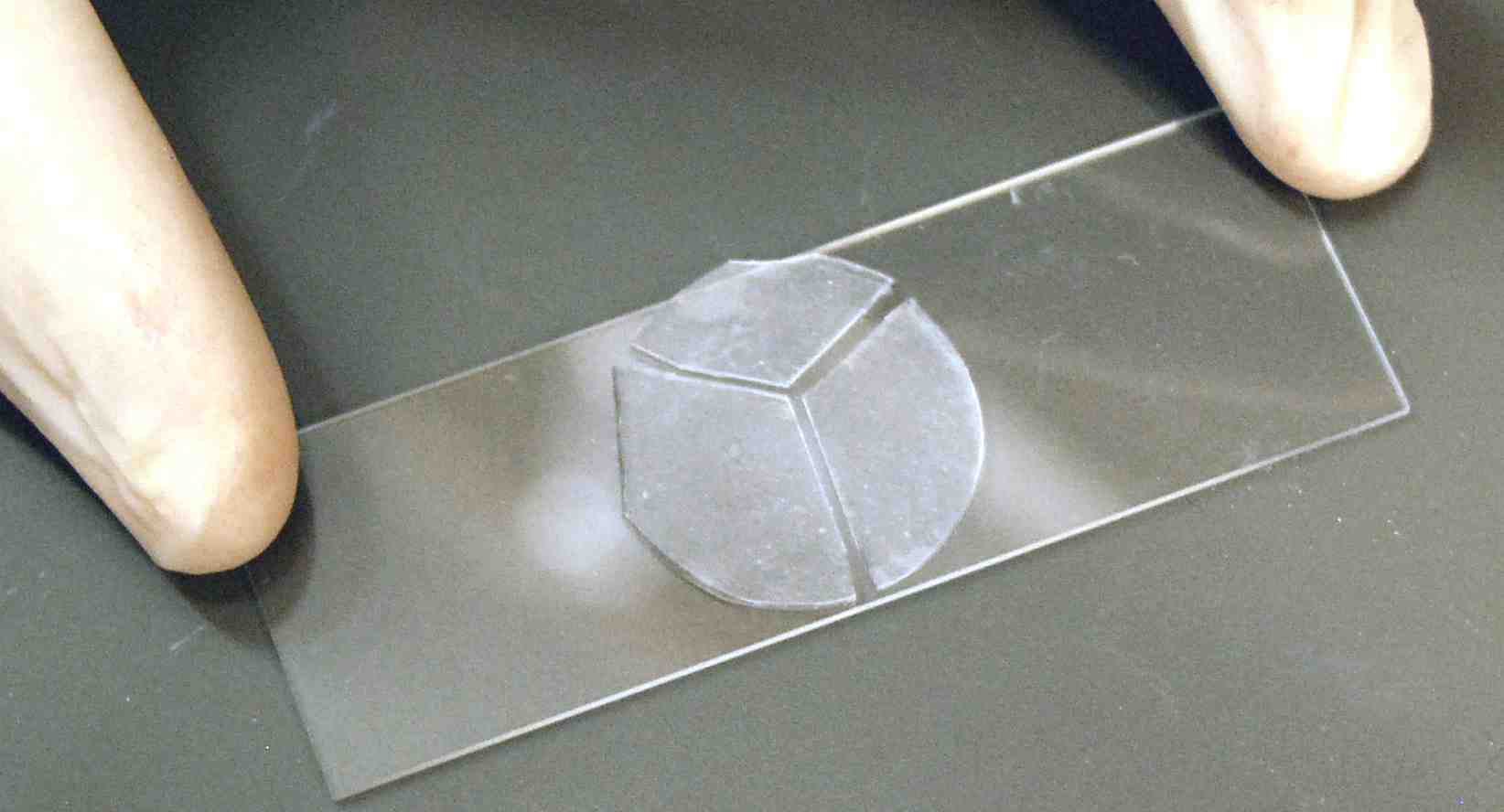 | Press on the coverslip |
| 13 |  | (Optional) Heat in the oven on 80-100^0^C for >10 minutes. |
| 14 |  | (Optional) Transfer immediately to the plasma cleaner, using only the vacuum for >5 minutes. After this devices can be stored in a clean environment indefinitely. |

Attaching Tubing

| **Step** | **Picture** | **Details** |
| --- | --- | --- |
| 1 | 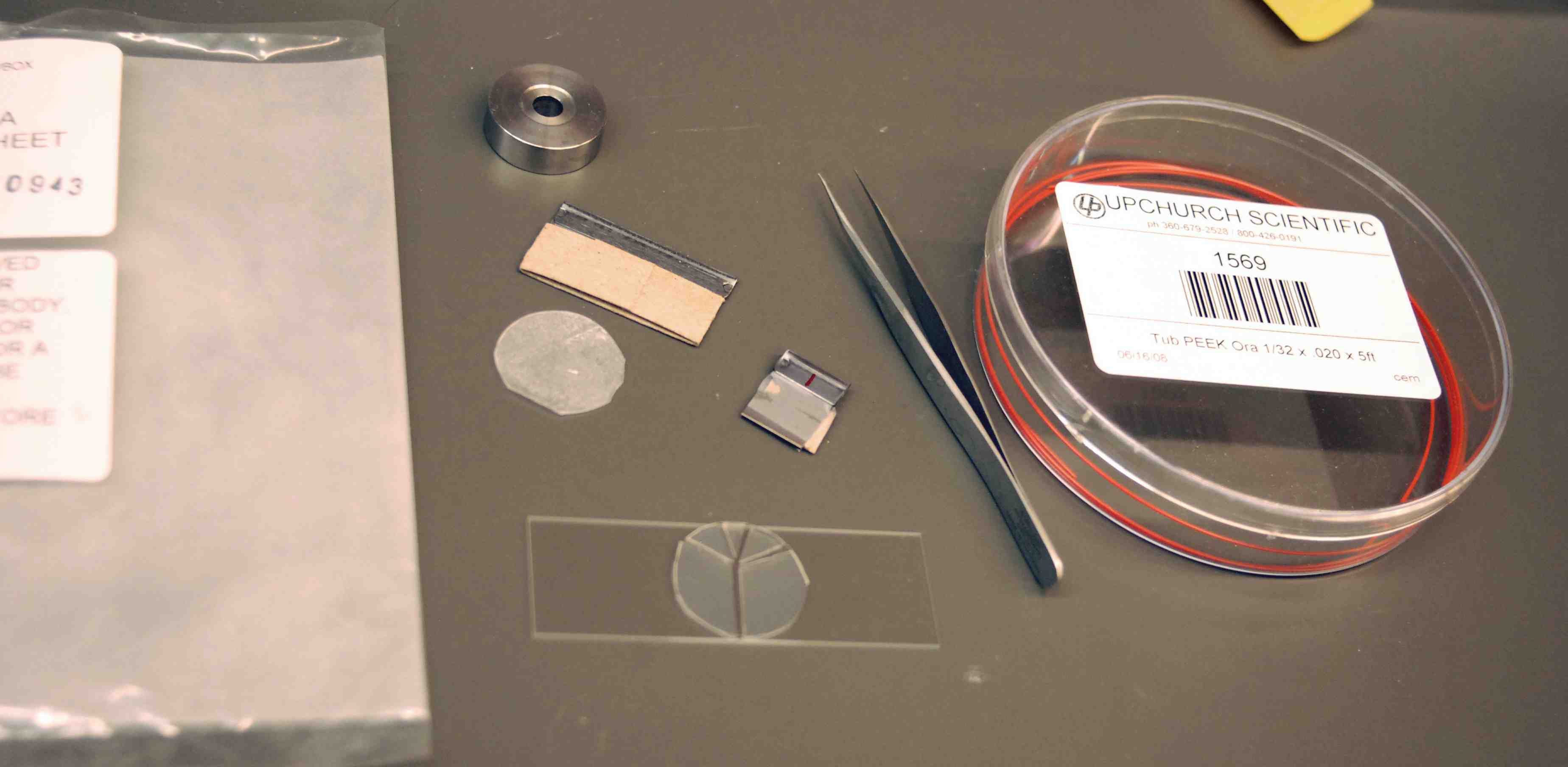 | Cut lengths of tubing to connect to your device (PEEK 1/32" OD, .020" ID). |
| 2 | 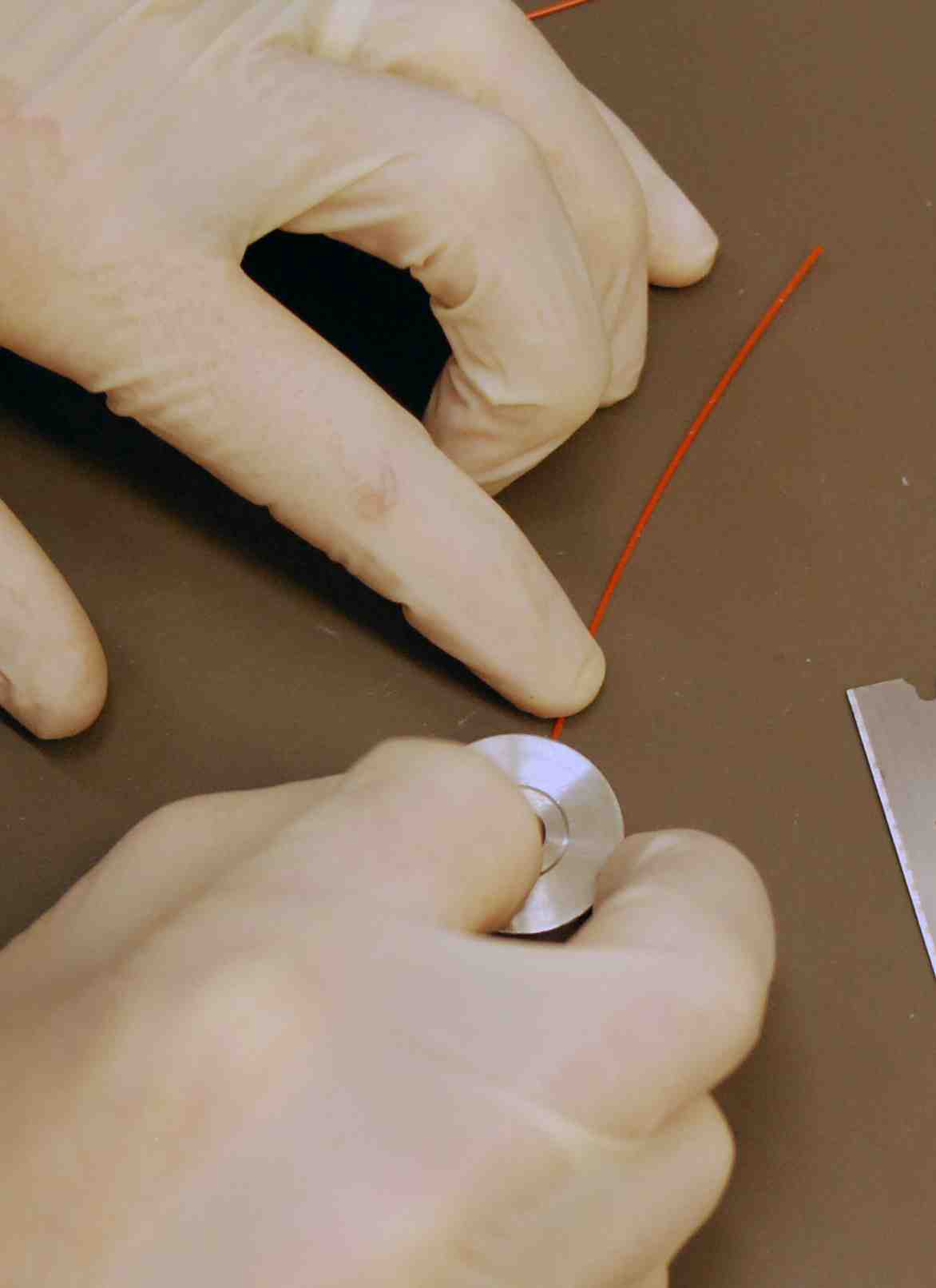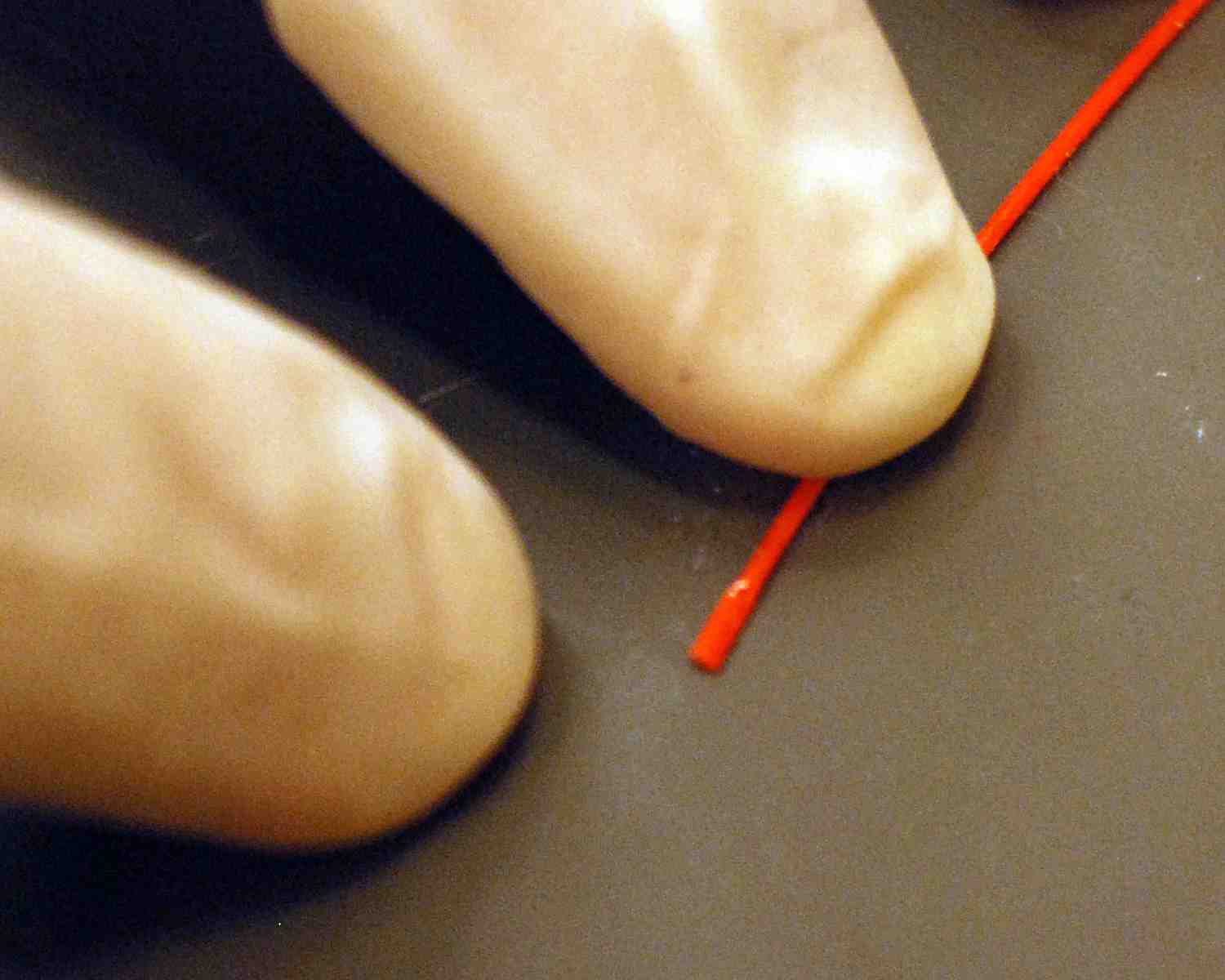 | Flatten one end of each piece of tubing using a metal block or other flat object. |
| 3 | 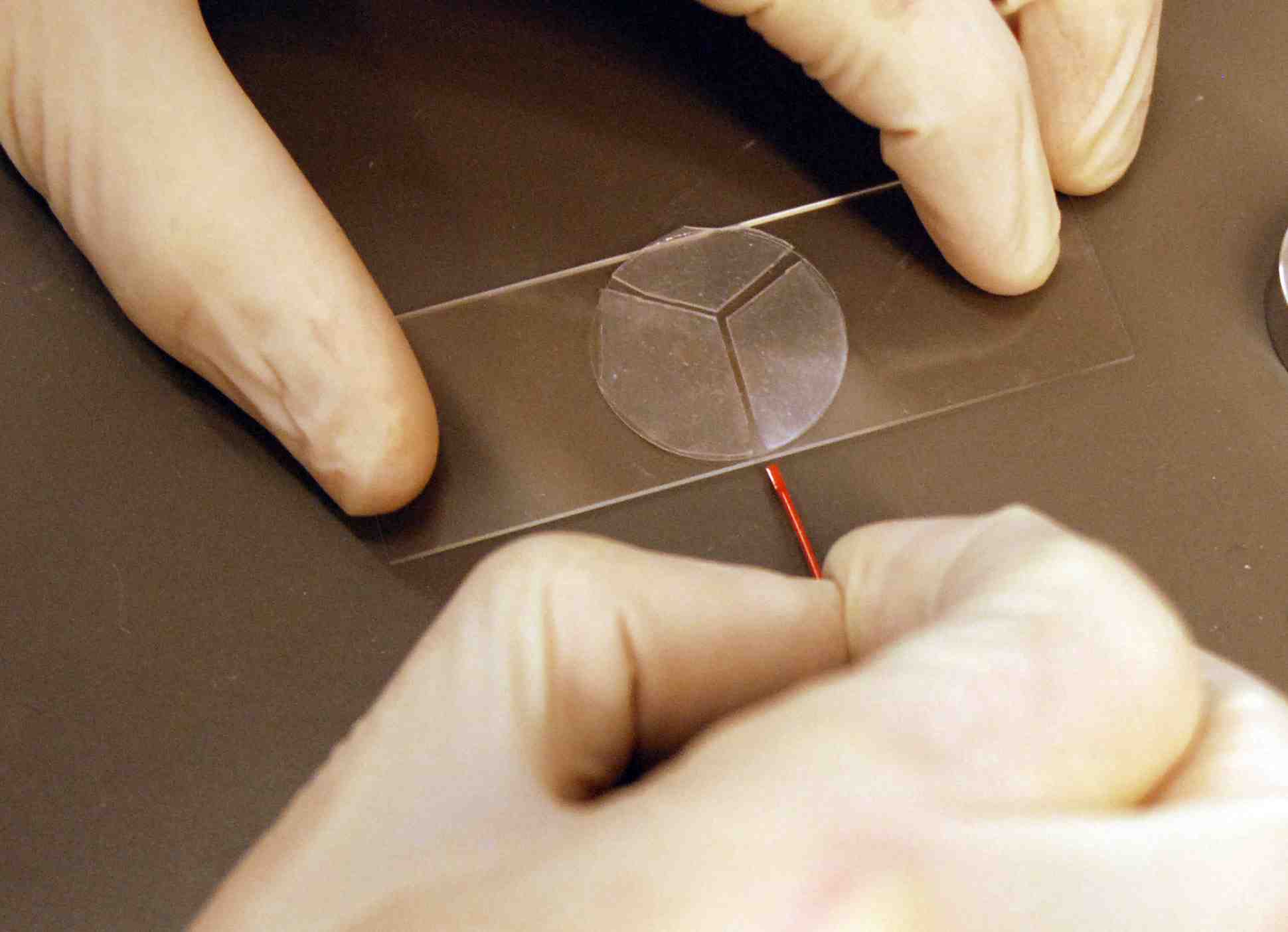 | Insert flattened end of tubing into device approximately 0.5cm. |
| 4 |  | Place device on Teflon block or other non-stick surface. Epoxy the tubing in place, making sure to seal all around each piece of tubing. 5-minute epoxy works well here. |
| 5 | 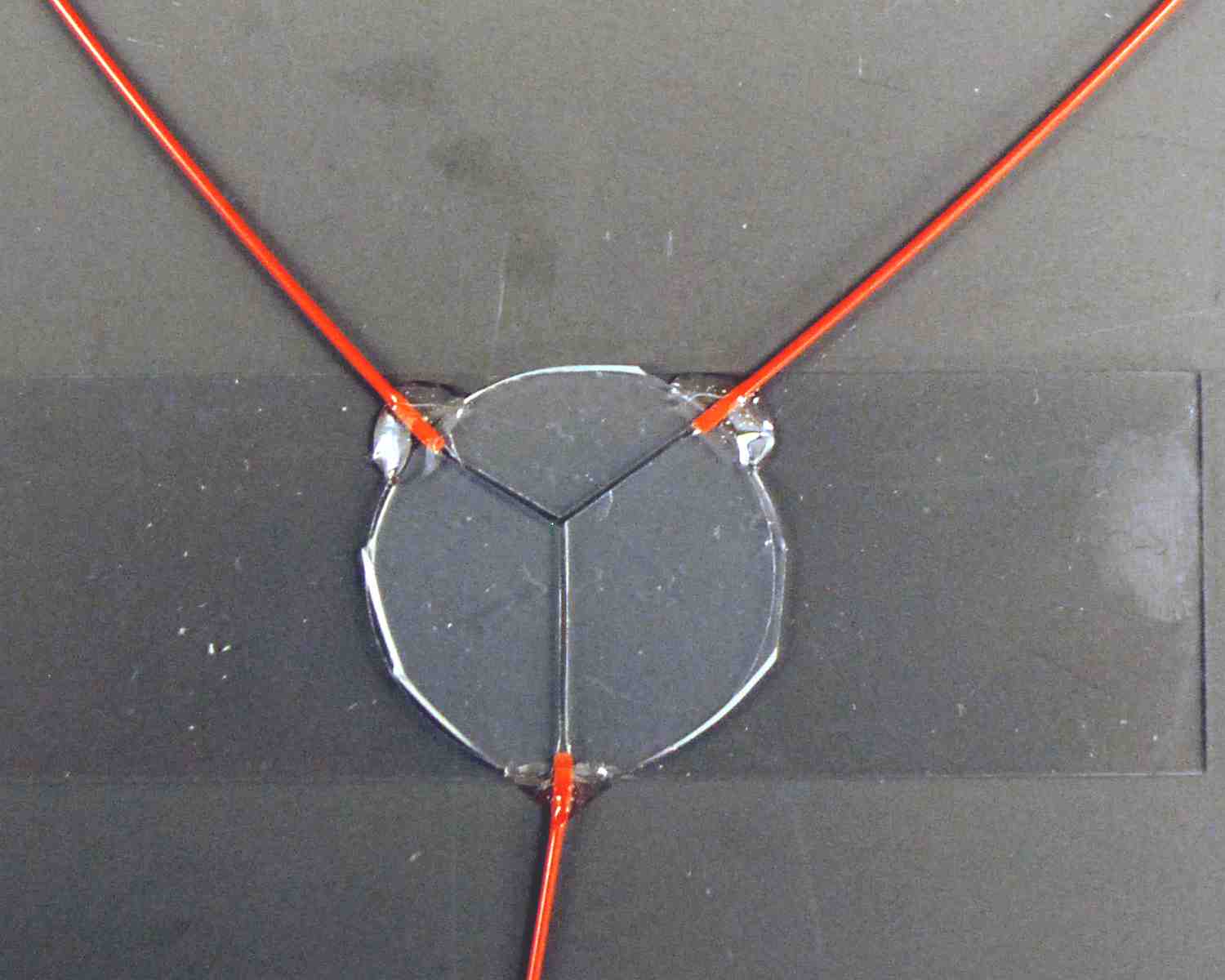 | Once epoxy has set, gently remove device from Teflon block. A razor blade is helpful to break any connection between the Teflon and the epoxy. The device is now complete. |

Connecting to Flow

| **Step** | **Picture** | **Details** |
| --- | --- | --- |
| 1 | 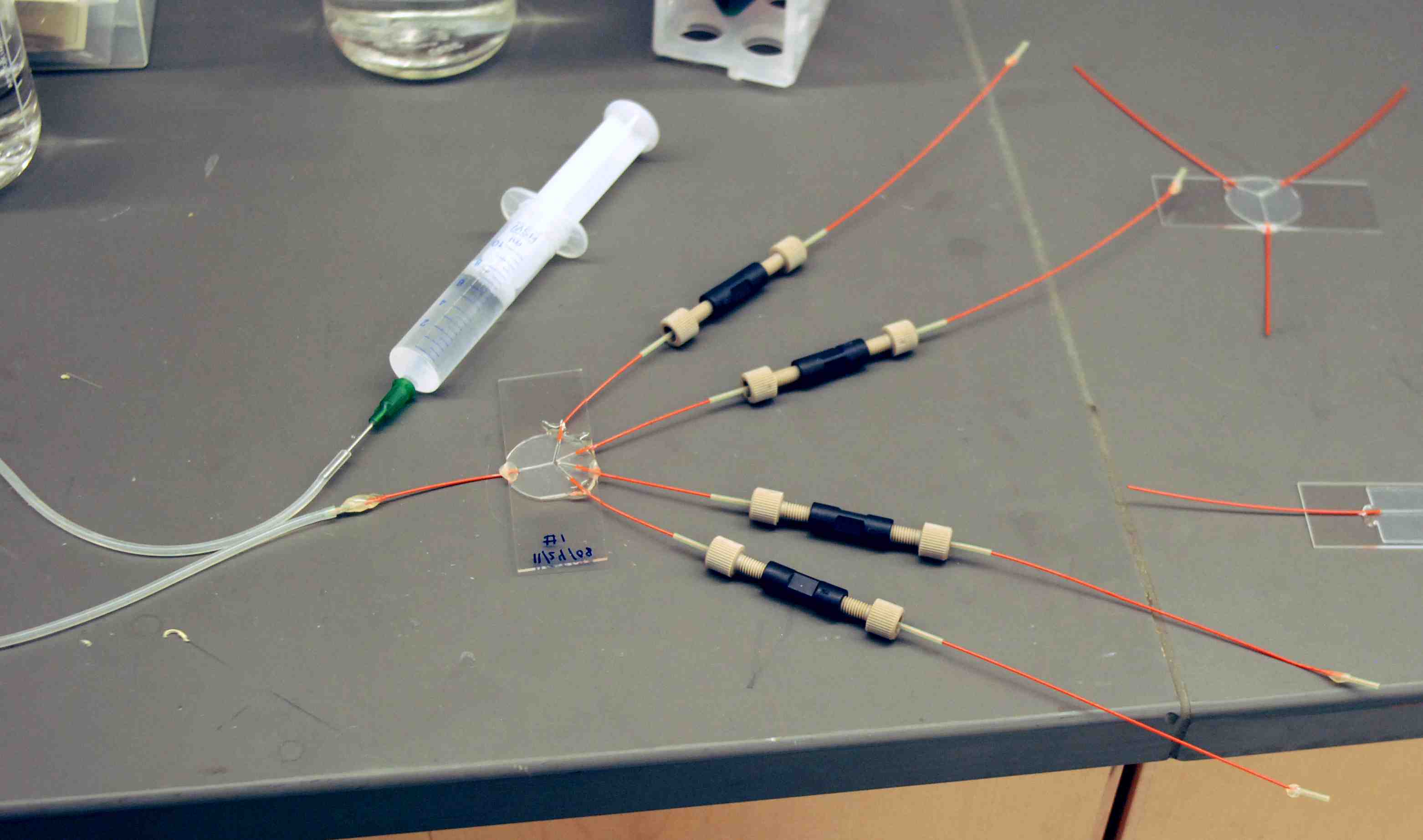  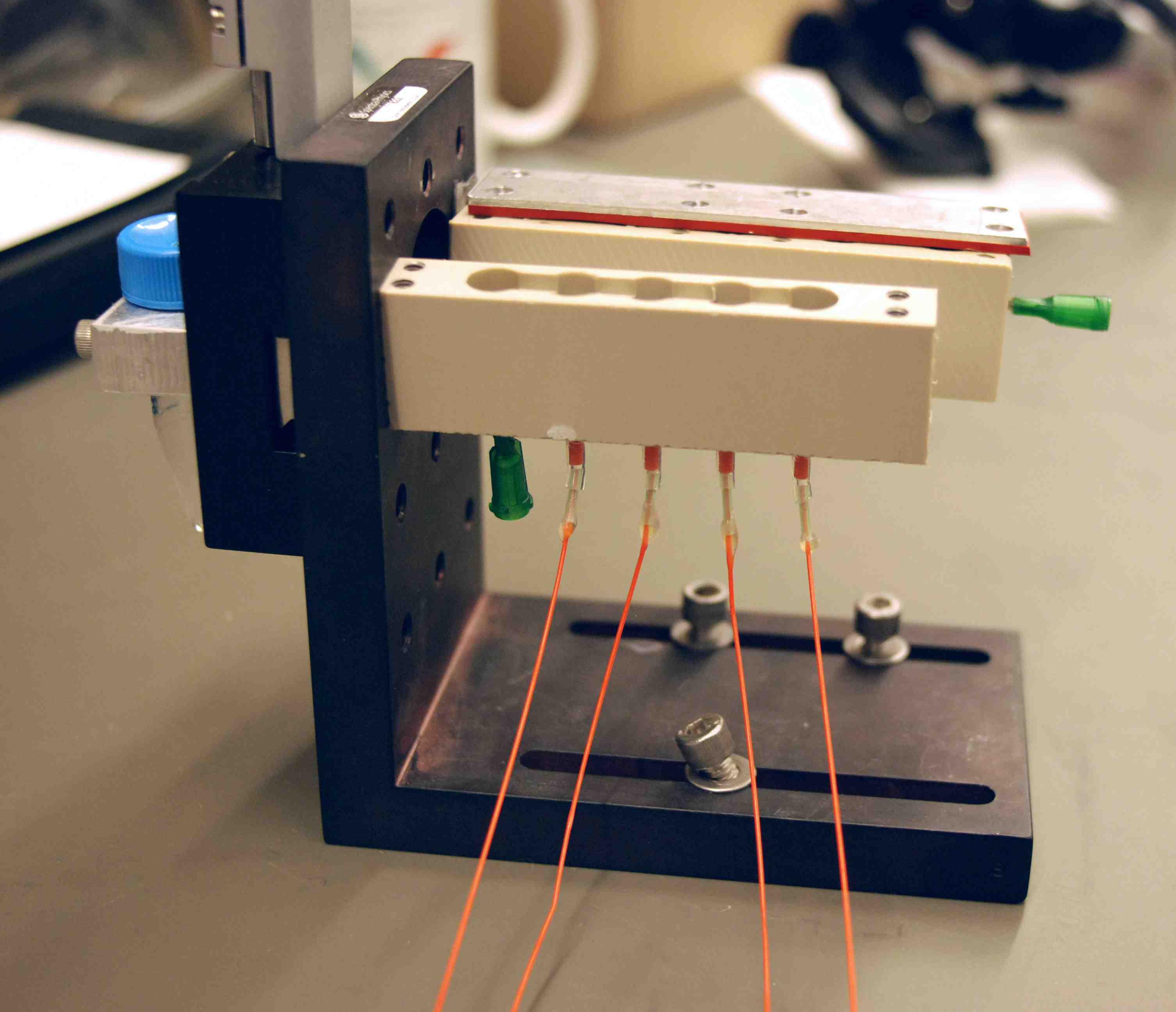 | Join device to flow control and reservoir system using Upchurch HPLC adaptors and tubing sleeves or pieces of silicon tubing. |
| 2 | 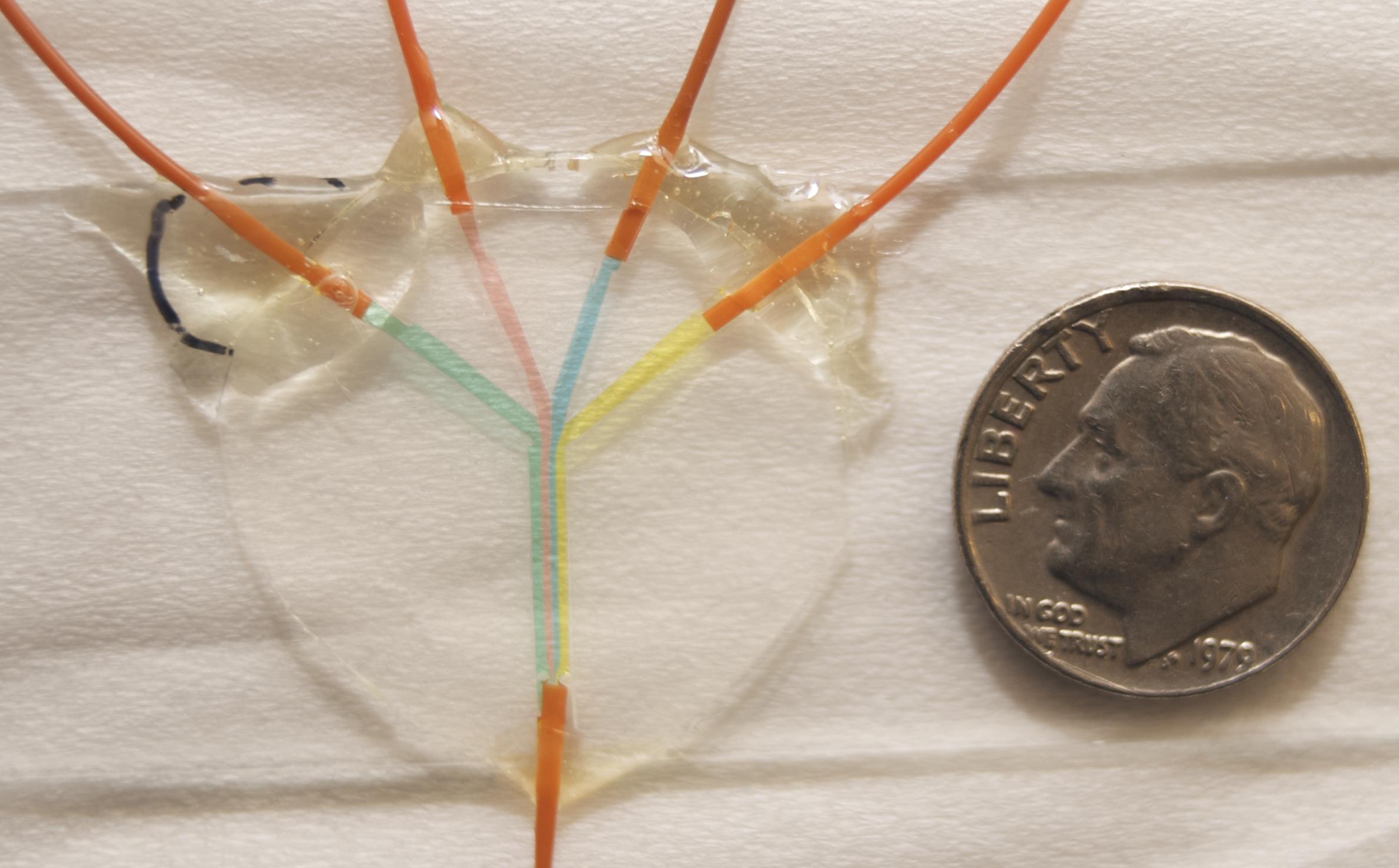 | Test your device for desired flow characteristics. Food color is useful for this, but devices must be thoroughly cleaned after use of food color. |

Types of Reservoir and Flow Control Systems

| **Picture** | **Description** |
| --- | --- |
| 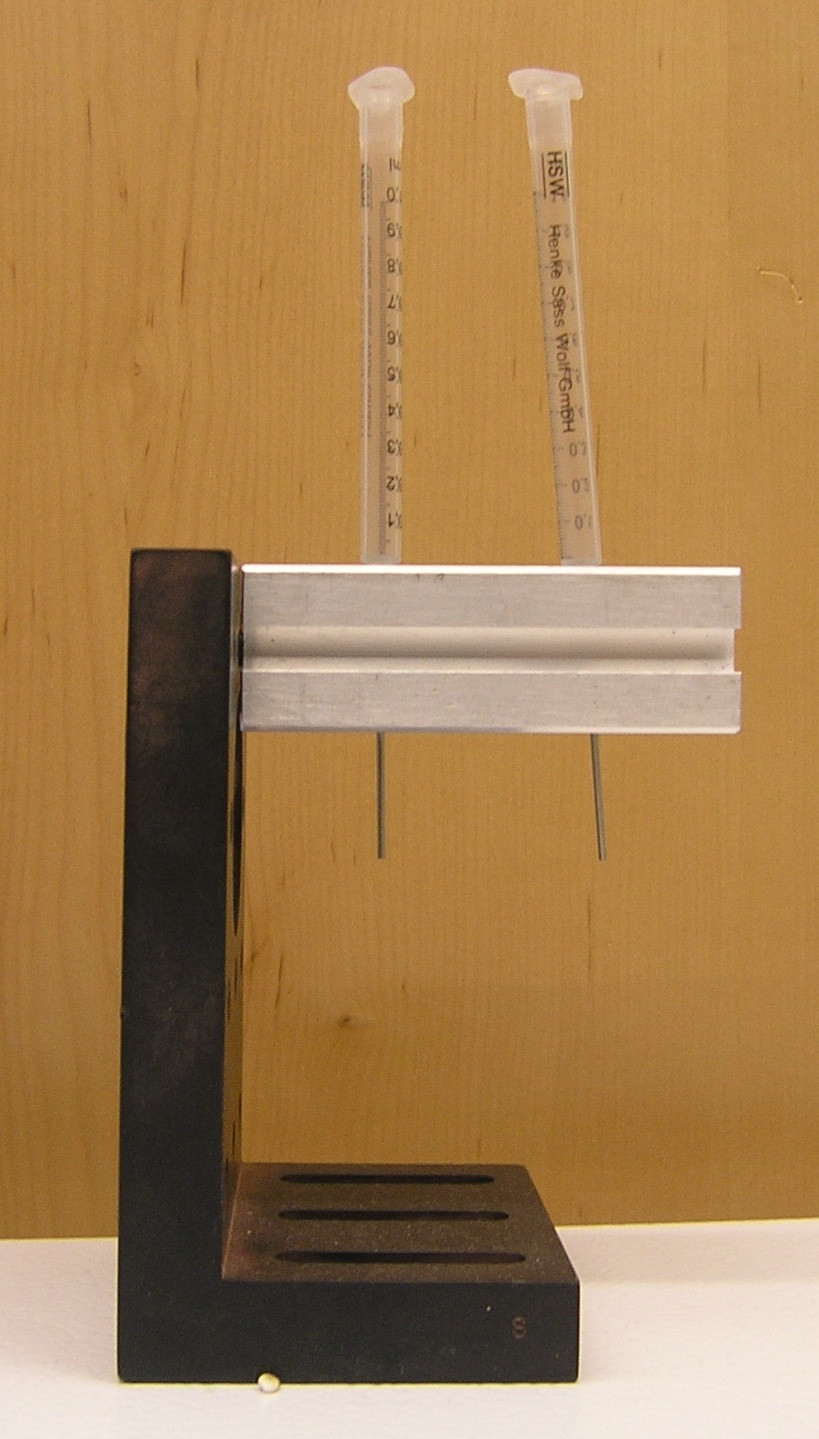 | Syringes with the plunger removed and set in a stationary block can be used as reservoirs for systems flowing by gravity. Having the output tube feed into a stationary collection reservoir is the easiest flow drive solution and is sufficient for many experiments. |
| 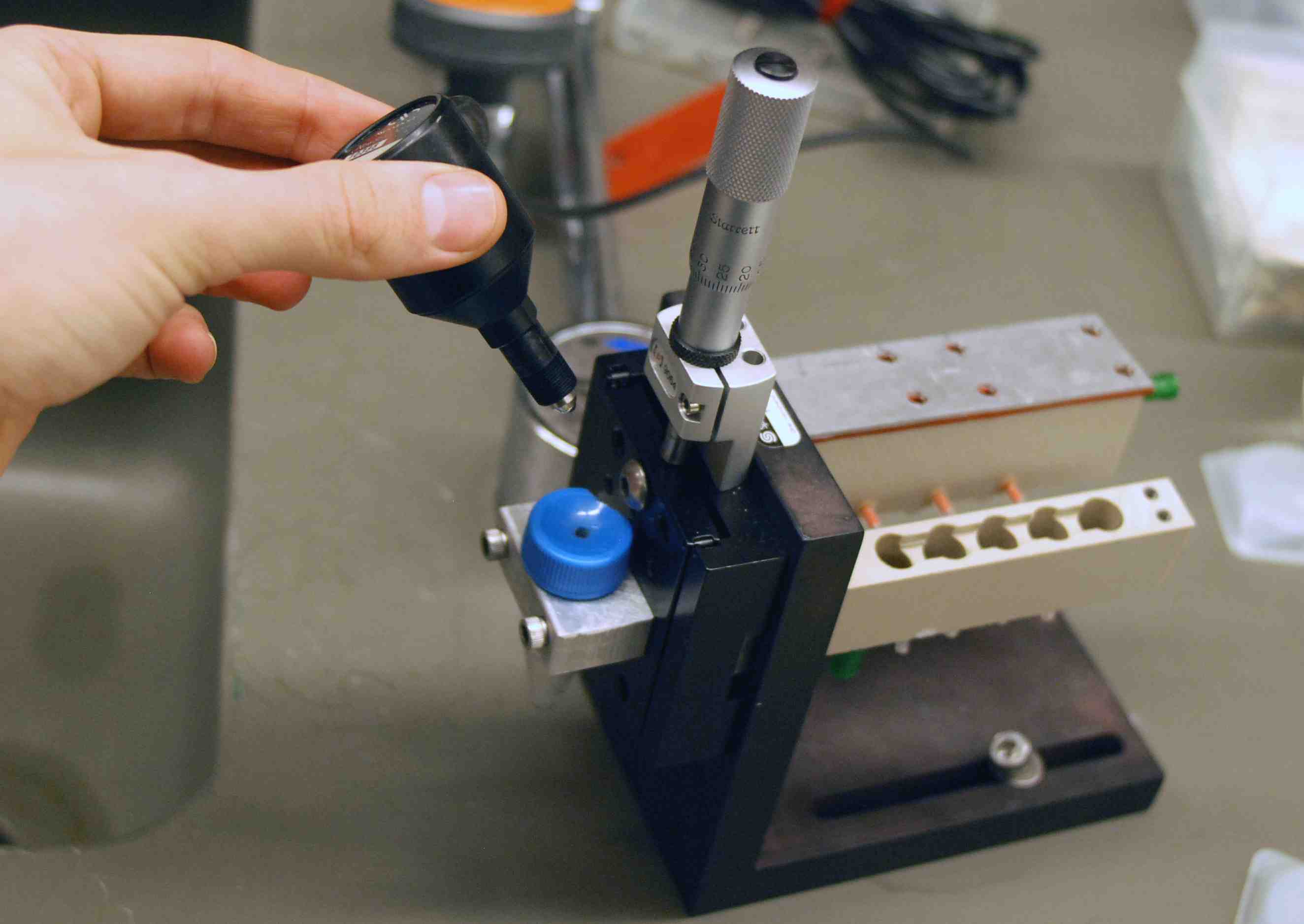 | Gravity flow rate can be adjusted by changing the height difference between the inputs and output. This can be done during experiments using a motorized or manual actuator attached to either the inputs or output. In the picture a manual or motorized actuator can be used to move the output reservoir (blue cap) up or down to adjust the flow rate. |
|  | Commercial pumps can be used to drive flow. The drawbacks to using these are that precise control at low flow rates is often difficult and individual pumps are often required for each reservoir. |
| 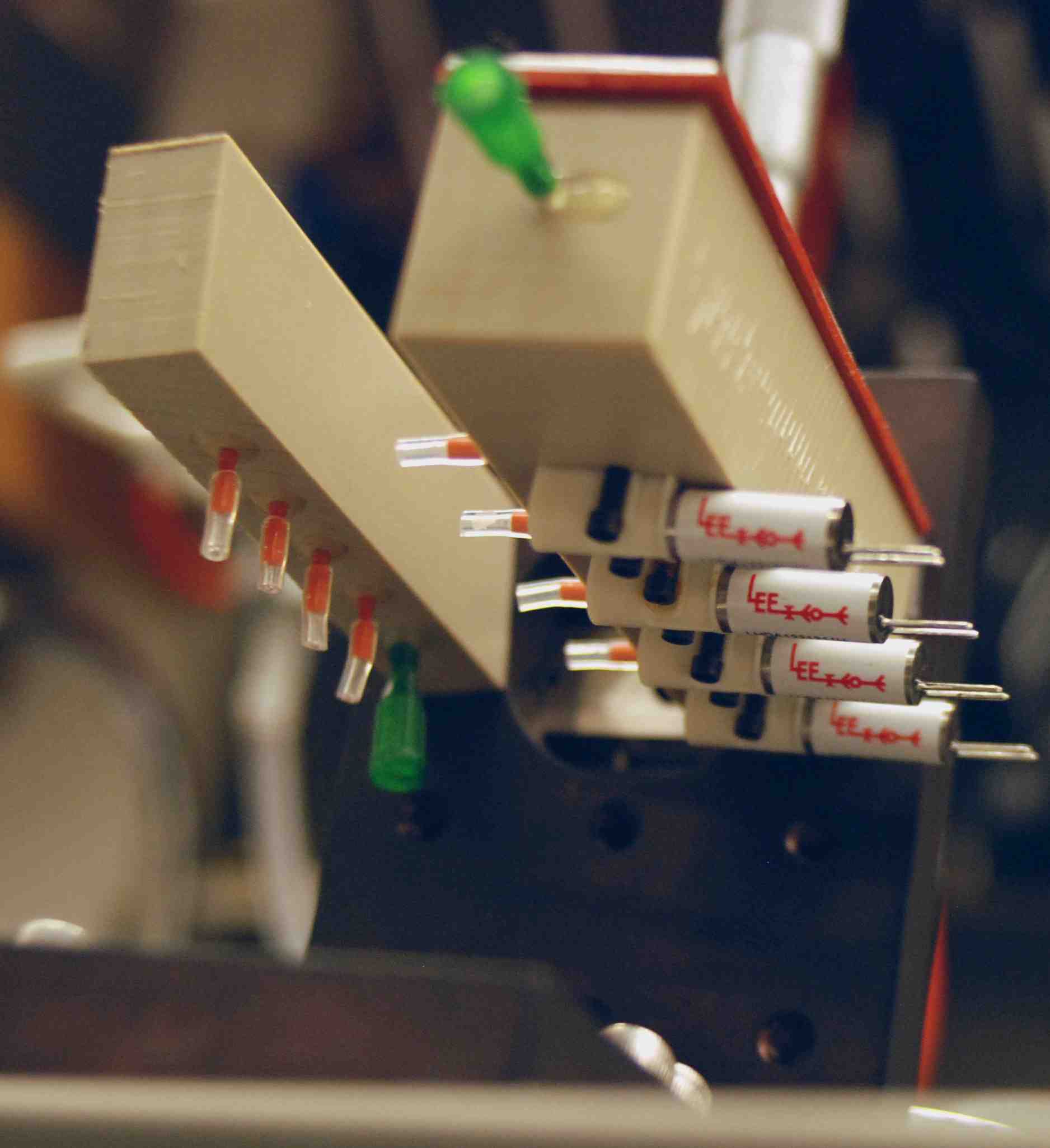 | Pressure manifolds can be used to drive flow and can be machined to specifications. They include reservoirs and an input for a pressurized gas. If your systems manifold seals well, adding a pressure gauge, an input regulator valve and a bleed valve are ideal for maintaining a constant and finely tunable flow rate. In the device shown a lid can seal the reservoirs and all outputs go through pressure rated valves. A nitrogen line is attached to the green syringe. This device allows a constant pressure to be built up in the chamber creating a constant pressure differential between the reservoirs and the output port and yielding consistent flow. The pictured manifold does not use a bleed valve and uses a regulated input line. |

Using the Device

**Important Note:**

• This is a basic protocol, each experiment will require adjustments.

• Make sure your device has been properly tested before using.

**Set up**

1. Connect device to reservoir and valve manifold(s).

2. Back prime with a syringe of water. Leave syringe attached.

3. Mount apparatus on the optical table.

4. Connect flow control.

5. Connect valve power to valves.

6. Put oil on the objective, place device, tighten down slide holders, put down condenser oil, lower condenser.

7. Adjust for Koehler illumination.

**Blocking**

1. Make 1ml of blocking solution and load into a syringe.

2. Flow into the device through the output port. note: you should see the level rise in all of the reservoirs, if you don't this means one of them is blocked, or the valve is closed.

3. Block for at least 2 minutes. Longer is ok.

**Focusing**

1. Turn the visual laser on and move the slide until the laser clips the edge of the channel.

2. Focus using the fluorescence or bright field. You need to get down near the coverslip.

3. Readjust Koehler.

**Loading Solutions**

1. Mix solutions in 1.7ml eppendorfs. Volume of solution is up to you. Between 200-1000ul seems to work well. Make sure you know the volume of your reservoirs before loading samples.

2. Close values

3. Pipet off excess water and blocking solution in reservoir left over from back priming.

4. Load samples.

5. Remove the syringe plugging the output port. Solutions can now flow freely.

6. Allow the dead volume of your system to flow out.

7. Adjust flow rate.

8. Begin experiment.

**Running Experiments**

• At this point you should be all set. If you are using gravity flow, remember it will slow down with time so you have to adjust the water column height regularly

• Set a timer for 20minutes. When it goes off, check your solution volumes in the reservoirs. Repeat this until you have a good idea of how long your solutions will last and when appropriate reloading times are.

• Reloading solutions will change the flow rate.

**When your experiment is over:**

1. Turn off the scope and flow drive equipment.

2. Reattach water syringe to output hose.

3. Remove device from stage.

4. Clean off condenser and objective oil from device.

5. Remove device mount from optical table.

6. Clean device

Cleaning Devices

**Important Notes**

• Clean your device after each day’s use

• These devices are reusable. Unless damaged or leaking you should not need to

make a new one.

• This is an empirically determined protocol. Adjust as you see fit.

• If your system has valves connected, make sure they are open.

• All solutions are added by back priming the system through the out port of the device.

This insures that each input receives equal washing.

• Wear gloves, you will be working with hazardous solutions.

**Cleaning**

| **Step** | **Picture** | **Details** |
| --- | --- | --- |
| 1 |  | Fill separate 12ml syringes with water, EtOH, and a mix of 80% 1M NaOH +20% EtOH |
| 2 |  | Attach the water syringe to the out port and rinse the device with water [~5mL]. |
| 3 | 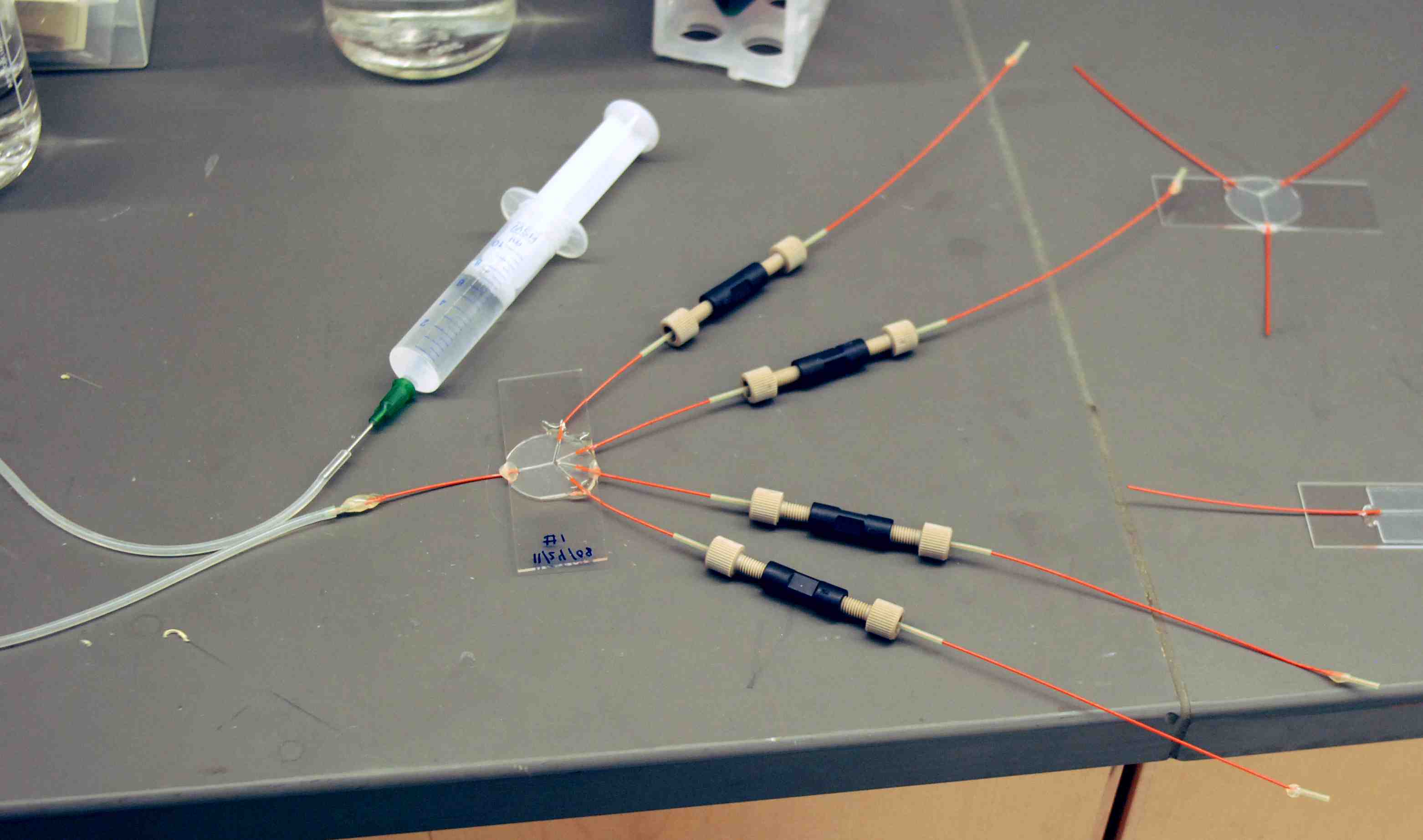 | Rinse with 80% NaOH +20% EtOH Solution. Push through about 5mL then let the device sit for 5 minutes with the NaOH solution inside. |
| 4 |  | Rinse with 5ml of EtOH. |
| 5 |  | Rinse with 5-20 ml water. |
| 6 |  | Leave the water syringe attached and store the device full of water. |
